# Dysphagia in Neurodegenerative Diseases: Aspects to be Considered when Seeking Quality of Life

**DOI:** 10.1055/s-0046-1818586

**Published:** 2026-03-11

**Authors:** Geraldo Pereira Jotz, Amanda dos Passos Sandrin, Arthur Viana Jotz, Vanessa Elias

**Affiliations:** 1 Universidade Federal do Rio Grande do Sul (UFRGS), Universidade do Vale do Sinos (UNISINOS), Collegium Oto-Rhino-Laryngologicum Amicitiae Sacrum (CORLAS), Rio Grande do Sul, Brazil; 2 Voice Institute, Porto Alegre, Brazil; 3 Pontifícia Universidade Católica do Rio Grande do Sul (PUCRS), Porto Alegre, Brazil; 4 Qualos Clinic, Porto Alegre, Brazil

## Introduction

Neurological diseases of diverse central and peripheral origins have a profound impact on swallowing. Among them, particular emphasis is placed on those caused by secondary effects and most frequently observed during the human aging process.

Although dysphagic conditions arise from distinct factors, they share common clinical characteristics that tend to result in dysphagia, with aging representing a fertile ground for its development. 1

Malnutrition, dehydration, and aspiration pneumonia negatively affect the health of individuals with oropharyngeal dysphagia, exerting a deleterious impact on their quality of life. Moreover, medical complications resulting from dysphagia may increase healthcare costs and length of hospital stay. 2 3 4

In this context, this editorial aims to highlight the neurodegenerative causes of dysphagia, emphasizing aspects already described in the literature that should be considered in Alzheimer's disease (AD), Parkinson's disease (PD), Multiple Sclerosis (MS), Amyotrophic Lateral Sclerosis (ALS), Dementia with Lewy Bodies (DLB), and Huntington's disease (HD). The objective is to draw attention to and contribute to improvements in the swallowing process and, consequently, to the quality of life of individuals affected by these conditions, in pursuit of excellence in human health.

## Relevant Aspects

Huntington's disease (HD) is a hereditary neurodegenerative disorder characterized by chorea, dementia, and emotional disturbances. It typically has adult onset and is associated with cognitive and psychiatric changes and autosomal dominant inheritance. 5 6 7 Oropharyngeal dysphagia in HD occurs because swallowing tends to be rapid and uncontrolled in these patients, due to disease-related signs and symptoms as well as impaired cognition. Additionally, uncoordinated tongue movements may occur, resulting in disorganized bolus propulsion. 8

Dysphagia affects patients as the disease progresses and becomes life-threatening in the later stages of HD due to subsequent respiratory complications. Dysphagic symptoms in patients with HD are observed during the anticipatory and oral preparatory phases, with difficulties in transporting food to the mouth, poor tongue control, and postural instability caused by chorea. Involuntary movements in the oral cavity may produce discoordination between the oral and pharyngeal phases. Furthermore, cognitive deficits may lead to a tendency to eat rapidly. 5

Dysphagia in Multiple Sclerosis (MS) is a relatively common symptom, with an incidence ranging from 24% to 65% of affected patients, depending on disease severity. 1 9 10 11 12 13 Its prevalence may increase significantly as MS progresses. Dysphagia negatively impacts patients' quality of life by increasing the risk of dehydration and aspiration 8 10 12 and is more severe in patients with brainstem involvement. 1 Aspiration pneumonia is the leading cause of death in patients with MS. The most common dysphagic signs include reduced tongue movement, delayed swallow initiation, and decreased laryngeal closure. The oral phase is more affected in cases of severe dysphagia. Upper esophageal sphincter dysfunction is common and becomes more frequent with disease progression. In addition, patients with MS often present deficits in motor control of the limbs and hands, resulting in feeding difficulties. 1

Dysphagia in patients with Amyotrophic Lateral Sclerosis (ALS) has a prevalence of ∼25%. 1 The tongue is markedly affected compared with other musculature, leading to significant impairment of the oral phase. 1 14 Dysphagia is one of the most frequent and debilitating comorbidities of ALS and is caused by progressive degeneration of the corticobulbar pathways and/or the motor nuclei of cranial nerves IX, X, XI, and XII, resulting in secondary impairment of pharyngolaryngeal contraction as well as tongue atrophy and dyskinesia. The vagus and glossopharyngeal nerves are primarily responsible for pharyngeal and laryngeal sensory and motor innervation; involvement of these cranial nerves leads to difficulty in elevating the soft palate, resulting in nasal regurgitation of food, as well as reduced laryngeal elevation, compromising airway protection during swallowing. 15 16 Patients with ALS may also present delayed triggering of the swallowing reflex, and the risk of aspiration is high. 1

In Parkinson's disease (PD), dysphagia is also a very common symptom, affecting more than 80% of individuals. 1 15 17 Swallowing alterations most frequently associated with PD involve the oral and pharyngeal phases, including impaired bolus formation, delayed swallow initiation, prolonged pharyngeal transit time with multiple swallows, 1 15 16 17 inefficient mastication, lingual tremor, and limited mandibular excursion. 18 It is noteworthy that early initiation of speech-language therapy allows for positive intervention aimed at slowing the progression of dysphagia in patients diagnosed with PD. What has already been lost cannot be recovered, but disease progression can be mitigated.

Dysphagia reduces quality of life, complicates medication intake, and leads to malnutrition and aspiration pneumonia, which is one of the leading causes of death in PD. 17 Although highly prevalent, patients often do not report swallowing difficulties due to the severity of motor manifestations.

Dementia is a condition characterized by progressive cognitive deterioration that affects daily functioning. 19 It is estimated that by 2040 there will be more than 81 million people with dementia worldwide. A hallmark of the advanced stage of dementia is loss of interest in eating and dysphagia. 1 20 The most common clinical manifestations include difficulties with chewing or food manipulation, food residue in the oral cavity—particularly solids and liquids—coughing during meals, 19 20 and the need for reminders to swallow. 19

In Alzheimer's disease (AD), dysphagia results not only from motor deficits but also from cognitive alterations, such as visual inability to recognize food, oral-tactile agnosia, and apraxia. 21 22 23 In advanced stages, dysphagia is estimated to affect ∼80% of patients with AD 23 and contributes to respiratory infections and hospitalizations. 24 According to the literature, pneumonia is the cause of death in up to 70% of patients. Poor oral hygiene is also an important factor, given the frequent aspiration of oral residues. 21 25 Alterations predominantly affect the oral and oropharyngeal phases, including delayed swallow reflex, reduced tongue–palate coordination, and decreased pharyngeal sensitivity. 21 22 26 Dysphagia in AD is associated with weight loss, malnutrition, and dehydration, which accelerate patient decline and negatively impact survival. 23 24 27 Concomitantly, dysphagia may lead to emotional discomfort during meals, increasing social isolation and further contributing to cognitive deterioration. 23 In advanced AD, the use of alternative feeding routes, such as feeding tubes, should be carefully evaluated, respecting patient and family wishes and ethical considerations. 21 23 24 Therefore, a multidisciplinary approach to dysphagia in AD is essential to ensure physical and emotional well-being for patients, families, and caregivers.

The prevalence of swallowing disorders ranges from 13% to 57% across different types of dementia. Among residents of long-term care facilities, dysphagia prevalence may reach up to 53%, and silent aspiration has been reported in up to 68% of these patients. Swallowing alterations vary according to the type of dementia. 19

Simple interventions, such as ensuring a calm environment and adequate time for meals, may be key factors in improving patients' quality of life. 23 Brainstem manifestations often precede cognitive changes. Swallowing biomechanics may be affected in all phases, notably disorganization of bolus formation and propulsion, delayed swallow initiation, reduced elevation and anterior movement of the hyoid bone and larynx, reduced pharyngeal contraction, residue in the valleculae and pyriform sinuses, silent penetration and aspiration, and impaired protection of the lower airway. 28

Early identification of dysphagia allows for appropriate decision-making, including referral to a multidisciplinary team, with particular emphasis on the roles of the otorhinolaryngologist, speech-language pathologist, and nutritionist, given that early symptoms commonly involve phonation and/or swallowing.

The speech-language pathologist assesses the individual's alertness for feeding, inspects oral cavity conditions (dentition and hygiene), adapts food consistencies according to bolus preparation and control, defines compensatory and postural strategies, and evaluates readiness to protect the airway and feed safely. 21 29 Studies demonstrate the progression of Alzheimer's disease and its detrimental effects on swallowing function, nutrition, and related quality of life, emphasizing the need for early dysphagia screening and timely nutritional interventions in clinical care. 22

## Final Comments

Neurodegenerative diseases are among the most important and most common causes of dysphagia in patients treated by otorhinolaryngologists. Speech-language therapy plays a crucial role in maintaining a certain level of quality of life within the context imposed by neurodegenerative disease, which remains difficult to manage pharmacologically. This represents an ongoing mission in caring for patients who are progressively losing control of their swallowing, with a focus on reducing the functional impact of dysphagia. The initiation of speech-language therapy, always applicable in these cases, should occur as early as possible.
